# Early preeclampsia is associated with persistence of hypertension 3 months postpartum in women: an observational study at a tertiary hospital in Brazil

**DOI:** 10.61622/rbgo/2025rbgo38

**Published:** 2025-07-15

**Authors:** Gustavo Moleiro Tallarico, Priscila Oliveira Barbosa, Luiz Sérgio Lima-Junior, Ricardo Carvalho Cavalli

**Affiliations:** 1 Universidade de São Paulo Ribeirão Preto Medical School Department of Gynecology and Obstetrics Ribeirão Preto SP Brazil Department of Gynecology and Obstetrics, Ribeirão Preto Medical School, Universidade de São Paulo, Ribeirão Preto, SP, Brazil.

**Keywords:** Hypertension, Postpartum period, Pre-eclampsia, Pregnancy, Blood pressure, Antihypertensive agents, Risk factors, Prevalence

## Abstract

**Objective:**

This study aimed to investigate the persistence of hypertension at three months postpartum in women who experienced preeclampsia during pregnancy.

**Methods:**

A prospective observational study at Hospital das Clínicas de Ribeirão Preto, Brazil, included 24 women. Blood pressure measurements and/or antihypertensive use were assessed, alongside risk factors such as body mass index (BMI), heart rate, lipid profile and C-reactive protein (CRP). Data on demographic, obstetric and lifestyle factors were also collected.

**Results:**

Out of 24 postpartum women enrolled in this study, 15 (62.5%) of participants remained hypertensive three months after delivery. Women with early-onset preeclampsia had a 2.36-fold increased risk of persistent hypertension at three months postpartum. No significant differences were found among risk factors for persistent hypertension.

**Conclusion:**

Our results indicate a high prevalence of persistent hypertension in women with preeclampsia in Brazil. The findings highlight the need for extended monitoring specially in early onset preeclampsia and targeted lifestyle interventions.

## Introduction

Preeclampsia affects 2% to 5% of pregnancies worldwide, contributing significantly to maternal and fetal morbidity and mortality, with around 70,000 maternal and 500,000 infant deaths annually.^(1,2)^ In Brazil, hypertensive disorders are one of the leading causes of obstetric deaths.^(3)^ Beyond the risks during gestation, preeclampsia is associated with the persistence of hypertension in the short term.

In addition to the negative outcomes during the gestational and neonatal period, preeclampsia is identified as an independent and significant risk factor for the development of cardiovascular diseases in the future.^(4)^ Women who have had preeclampsia face a substantially elevated risk – two to four times higher – of developing chronic hypertension compared to those whose pregnancies occurred without complications, even 10 to 20 years after delivery.^(4)^ Recent evidence from a systematic review also indicates that women who experienced preeclampsia during pregnancy are more likely to develop hypertension in short term postpartum, within up to 6 months.^(5)^

The International Society for the Study of Hypertension in Pregnancy (ISSHP) establishes guidelines for the classification, diagnosis and treatment of preeclampsia at an international level.^(6)^ According to this clinical guideline, blood pressure is expected to return to normal within three months postpartum.^(6)^ However, studies from low- and middle-income countries report persistent hypertension in 28% to 39% of women beyond this period.^(7,8)^ The ISSHP guideline may not account for the variability in preeclampsia severity, particularly in early versus late-onset cases, and often lack specific recommendations for extended follow up.

In Brazil, data on the persistence of hypertension after preeclampsia are scarce, making it essential to investigate this issue in diverse populations. Additionally, obstetric, metabolic and inflammatory factors – such as body mass index (BMI), lipid profile and C-reactive protein (CRP) – appears to be associated with persistent hypertension.^(9)^ Therefore, this study aims to evaluate the persistence of hypertension in women with a history of preeclampsia after three months postpartum and to investigate the association between persistent high blood pressure and key metabolic and inflammatory biomarkers. Understanding these relationships is crucial for improving postpartum following-up strategies, preventing long-term cardiovascular complications, and optimizing maternal health outcomes.

## Methods

The present study adopted a prospective cohort observational design following the Strengthening the Reporting of Observational Studies in Epidemiology (STROBE) guidelines. Participants were selected during July 2021 to March 2024 from a sample of new cases at the Hospital das Clínicas de Ribeirão Preto. The study period extended to three months postpartum.

Participants were identified and recruited during their hospital stay in the immediate postpartum period. The inclusion criteria were women who had recently given birth and had been diagnosed with preeclampsia. The study included postpartum women who had experienced preeclampsia in any of its forms, including mild or severe preeclampsia, as well as early-onset, late-onset, or postpartum preeclampsia.

Preeclampsia was defined according to the criteria of the International Society for the Study of Hypertension in Pregnancy (ISSHP).^(6)^ Mild preeclampsia was characterized by new-onset hypertension (≥140/90 mmHg) after 20 weeks of gestation, accompanied by proteinuria (≥300 mg/24h) or other signs of organ dysfunction. Severe preeclampsia was diagnosed in cases where blood pressure reached ≥160/110 mmHg, or when there were severe features, such as thrombocytopenia, renal insufficiency, impaired liver function, pulmonary edema, or neurological symptoms. Early-onset preeclampsia was defined as occurring before 34 weeks of gestation, while late-onset preeclampsia developed at or after 34 weeks. Postpartum preeclampsia was diagnosed when hypertension and associated symptoms first appeared after delivery.

As part of standard care for preeclampsia, some participants received antihypertensive medications upon diagnosis. Additionally, some women may have used aspiring for preeclampsia prevention during pregnancy, as per medical recommendation. Oral antihypertensive therapy could be continued for women at hospital discharge, if necessary.

Exclusion criteria included women with chronic hypertension – defined as hypertension (≥140/90 mmHg) diagnosed before pregnancy of before 20 weeks of gestation – as well as those who did not attend any of the scheduled follow-up appointments during the three months postpartum period.

Data were collected on age, parity (nulliparity or multiparity), obstetric history, including the gestational age at the time of preeclampsia diagnosis and whether it was early-onset or late-onset. Biochemical tests performed at the time of the preeclampsia diagnosis were also assessed. The study documented the use of antihypertensive medication as well as other medications prescribed to unrelated to blood pressure management. Additionally, information about lifestyle habits, smoking and alcohol use was also included.

The primary outcome of the study was the persistence of hypertension three months after delivery, determined by systolic blood pressure (SBP) ≥ 140mmHg or diastolic blood pressure (DBP) ≥ 90mmHg, or the use of antihypertensive medication at the time. Blood pressure was measured at the three months postpartum using a standard protocol. Patients were seated comfortably with the sphygmomanometer cuff placed on the upper left arm. Blood pressure was measured three times with a 2-minute interval between each measurement, and the average was calculated from the results obtained at each appointment.

In addition to blood pressure, risk factors associated with persistent hypertension were also measured. Weight was assessed using a digital scale, and height was measured using a stadiometer attached to the scale; body mass index (BMI) was calculated from these measurements. Blood samples were collected to assess total cholesterol, LDL-cholesterol, HDL-cholesterol, triglycerides and C-reactive protein. Fasting was not required for the blood tests, which were performed in a laboratory by trained nurses using a vacuum blood collection device and a BD Vacutainer® serum tube to minimize handling and ensure accuracy. Medical records in relation to previous diseases were also assessed.

Quantitative variables were summarized using measures of central tendency and dispersion. Qualitative variables were presented using absolute and relative frequencies. To compare qualitative variables between groups (normotensive vs persistent hypertension), the chi-square test was used, while Mann-Whitney was applied for quantitative variables. Relative risks were calculated for differences found in the chi-square and t-test. To check if the data fit the models well, residual analysis was performed using scatter plots between predicted and observed values histograms and Q-Q plots. The analyses were implemented using SAS version 9.4.

The study was approved by the Research Ethics Committee of the Hospital das Clínicas of Ribeirão Preto Medical School 4.879.272 (*Certificado de Apresentação de Apreciação Ética:* 47225021.6.0000.5440). The research was conducted only after the participants signed Informed Consent Form (ICF). Participants were assured that they could refuse or withdraw from the study at any time without penalty.

## Results

We invited 78 women to participate in the study, of whom 45 were included and signed the informed consent form. However, 21 participants were excluded due to failure to return for follow-up three months postpartum. Therefore, our study encompassed 24 postpartum women [median age of 33.5 years, IQR-23.5 to 35.5], with persistent hypertension identified in 62.5% (n = 15) at three months follow-up. As detailed in table 1, significant differences were observed between the normotensive women and those with persistent hypertension: gestational age at diagnosis differed by 8 weeks (p = 0.0133), onset of preeclampsia (p = 0.0082), and delivery timing showed a 2-week preterm disparity (p = 0.0305). Although maternal age did not differ significantly between groups (p = 0.0931), women with persistent hypertension were older than normotensive women. Most of women were multiparas (58.3%) and cesarean section predominating (79.2% of cases). While 33.3% of participants had eclampsia imminence and 37.5% required magnesium sulfate therapy, we did not observe differences between the groups. Notably, 100% of women with persistent hypertension required antihypertensive medication at discharge, 44.4% in the normotensive group (p = 0.0012) (Table 2). Lifestyle assessments revealed concurrent tobacco use in 33.3% of women and alcohol exposure in 25%. We conducted a relative risk analysis, early-onset preeclampsia conferred a 2.36-fold increased risk of persistent hypertension (95% CI: 1.16-4.82), though biochemical profile – including uric acid, lactate dehydrogenase, hepatic transaminase, renal function markers, and platelet counts – showed no significant differences between groups (Table 3). Additionally, the prescription of antihypertensive medication at discharge was linked to a 4.75-fold increased risk of persistent hypertension (95% CI: 1.98-11.35).

**Table 1:** Characteristics of women who attended the study

Characteristics		Total (n=24) n(%)	Normotensive at 3 months (n=9; 37.5%) n(%)	Persistent hypertension at 3 months (n=15; 62.5%) n(%)	p-value
Maternal age (years)		33.5(23.5-35.5)	28(21-33)	35(25-37)	0.0931
Gestational age at diagnostic (weeks)		33(28-37)	36.5(34-37.5)	28(28-35)	0.0133
Onset preeclampsia					
	Early	11(45.8)	1(11.1)	10(66.7)	0.0082
	Late	13(54.2)	8(88.9)	5(33.3)	
	Gestational age at delivery (weeks)	36(33.5-37.5)	38(35-39)	36(30-37)	0.0305
Gravidity					
	Primigravida	14(58.3)	5(55.6)	9(60)	0.8307
	Multigravida	10(41.7)	4(44.4)	6(40)	
Mode of delivery					
	C-section	19(79.2)	8(88.9)	11(73.3)	0.3636
	Vaginal delivery	5(20.8)	1(11.1)	4(26.7)	
Eclampsia imminence					
	Yes	8(33.3)	2(22.2)	6(40)	0.3711
	No	16(66.7)	7(77.8)	9(60)	
Magnesium sulphate					
	Yes	9(37.5)	2(22.2)	7(46.6)	0.2311
	No	15(62.5)	7(77.8)	8(53.3)	
Anti-hypertensive medication at discharge					
	0	5(20.8)	5 (55.6)	0 (0)	0.0012
	≥ 1	19(79.2)	4 (44.4)	15 (100)	
Smoking					
	No	16 (66.7)	6 (66.7)	10 (66.7)	1.000
	Yes	8 (33.3)	3 (33.3)	5 (33.3)	
Alcohol consumption					
	No	18 (75)	7 (77.8)	11 (73.3)	0.8077
	Yes	6 (25)	2 (22.2)	4 (26.7)	

**Table 2:** Risk factors for persistent hypertension at three months postpartum

	Normotensive at 3 months (n=9; 37.5%) n(%)	Persistent hypertension at 3 months (n=15; 62.5%) n(%)	p-value
BMI ≥ 25 kg/m²	8(88.9)	13(81.2)	0.6170
Diabetes Mellitus	0(0)	4(26.7)	0.0897
Cholesterol > 190 mg/dL	3(33.3)	4(26.7)	0.7279
HDL < 40 mg/dL	3(33.3)	5(33.3)	1.000
LDL > 100 mg/dL	3(33.3)	8(53.3)	0.3411
Triglycerides > 175 mg/dL	0(0)	1(6.7)	0.4288
C-Reactive Protein			
Normal < 0.4 mg/dL	8(88.9)	8(53.3)	0.1546
Elevated 0.4 to 1.0 mg/dL	1(11.1)	3(20)	
Very Elevated > 1.0 mg/dL	0(0)	4(26.7)	

BMI: body mass index

**Table 3:** Biochemical profile of preeclampsia women included in the study

	Normotensive at 3 months (n=9; 37.5%)	Persistent hypertension at 3 months (n=15; 62.5%)	p-value
Uric Acid (mg/dL)	6.25 (3.54-6.83)	5.76 (5.45-7.19)	0.6892
AST (mg/dL)	18.46 (15.59-20.79)	19.61 (14.65-26.64)	0.8741
Urea (mg/dL)	24.33 (12.05-33.41)	23.28 (17.68-26.54)	08121
LDH (mg/dL)	271.9 (239.39-292.32)	263.03 (228.55-354.69)	0.8741
Creatinine (mg/dL)	0.57 (0.47-0.88)	0.68 (0.58-0.85)	0.6345
UPCR (μg/mg)	2199.01 (366.41-4359.01)	782.25 (438.02-1969.49)	0.9671
Plateletes (x10³/uL)	270 (217-342)	218 (158-270)	0.1909

AST: aspartate aminotransferase; LDH: lactate dehydrogenase; UPCR: urine protein-to-creatinine ratio

Table 2 presented conventional risk factors related to persistent hypertension. We did not find any differences between the risk factors, including BMI ≥ 25 kg/m^2^, diagnosed of diabetes mellitus, alteration of lipid profile and C-reactive protein.

## Discussion

Our study assessed the persistence of hypertension in women with preeclampsia, with follow-up extending to three months postpartum. To our knowledge, this is the first Brazilian study to specifically evaluate sustained hypertension in preeclamptic women. We found that 62.5% of participants remained hypertensive at three months postpartum, with early-onset preeclampsia conferring a 2.36-fold increased risk of persistent hypertension (95% CI: 1.16–4.82). These results highlight the need of systematic postpartum surveillance for women with preeclampsia, with enhanced monitoring protocols for those exhibiting high-risk features such as early-onset preeclampsia. Furthermore, targeted lifestyle interventions are fundamental to facilitate the body's return to normality after hypertensive disorders during pregnancy and to reduce the risk cardiovascular diseases later in life.

The incidence of new cases of hypertension in our study was higher than in similar studies with similar follow-up periods. Lugobe et al.^(8)^ carried out a study in Uganda, evaluating women with hypertensive disorders of pregnancy, found an incidence of 40%. A multicenter study in Tanzania reported that 41.1% of women who experienced gestational hypertension, preeclampsia, or eclampsia during pregnancy had persistent postpartum hypertension.^(10)^ Geographically closer to Brazil, a study in Cuba assessing women with preeclampsia found that 27.8% had persistent hypertension.^(7)^ We hypothesize that the elevated rate of persistent hypertension in our study may linked to factors such as advanced maternal age – women with persistent hypertension were older than 30 years, a threshold identified in prior studies as a risk factor for postpartum hypertension in preeclamptic women^(11,12)^ – as well modifiable lifestyle factors, with 33.3% and 25% of participants reported smoking and alcohol consumption, respectively.

Our research demonstrated that women with early-onset preeclampsia face a twofold increased risk of developing persistent hypertension, aligning with existing scientific literature.^(7,13,14)^ A study by Fajardo Tornes in Cuba similarly identified early-onset preeclampsia as a predictor of persistent hypertension.^(7)^ Early-onset preeclampsia is recognized as a more severe and aggressive variant of the condition, potentially resulting from placental dysfunction.^(15)^ The inability of the cardiovascular system to adapt during pregnancy, combined with early-onset preeclampsia, may result in lasting vascular alterations, increasing susceptibility to persistent hypertension and other cardiovascular issues later in life.^(12)^

Our findings further revealed that women discharge4d with antihypertensive prescriptions were nearly five times more likely to develop persistent hypertension compared to their normotensive counterparts. This aligns with a study conducted in Vietnam, where women prescribed antihypertensive medications postpartum exhibited a higher likelihood of persistent hypertension.^(16)^ These results underscore the importance of studies like ours in identifying women at greater risk of persistent hypertension, ensuring they are closely monitored and provided with targeted lifestyle guidance.

The ideal clinical management of preeclampsia and its postpartum effects remains unclear. According to International Society for the Study of Hypertension in Pregnancy (ISSHP), women should be reassessed three months postpartum to verify the normalization of blood pressure and other conditions.^(6)^ This recommendation assumes that most clinical conditions are resolved within this time frame. However, our study suggests this timeframe may inadequately capture persistent cardiovascular adaptations in high-risk women, as indicated by other studies.^(12,17,18)^ Specifically, 62.5% of our cohort exhibited sustained hypertension at the three-month mark, disproportionately affecting women with early-onset preeclampsia (RR 2.36, 95% CI: 1.16–4.82).

Our study's main strength includes the assessment of postpartum women using conventional risk factors in patients with preeclampsia and being the first study to evaluate persistent hypertension postpartum in Brazil. However, the study has some limitations, such as low patient adherence, potentially due to challenges in accessing the hospital with a newborn and the impact of the COVID-21 pandemic. Additionally, the hospital where participants were identified is a tertiary care reference center, primarily managing complicated cases, which may limit the generalizability of findings to other healthcare settings. Further studies are needed to evaluate the incidence of persistent hypertension across different levels of healthcare to provide a more comprehensive understanding of this condition.

## Conclusion

Persistent hypertension was very common three months postpartum, suggesting that the cardiovascular system remains affected by a history of preeclampsia, particularly in women with early-onset preeclampsia. Our study provides valuable insights into the postpartum period, emphasizing the need for extended monitoring of women with a history of early onset of preeclampsia.
